# Soft Tissue Benign Hand Masses in the First Web Space: A Retrospective Case Series

**DOI:** 10.7759/cureus.37847

**Published:** 2023-04-19

**Authors:** Ehab S Saleh, Alex F De Carvalho, Sazid Hasan, Amr Abdelgawad, Ahmed M Thabet, Moheb S Moneim

**Affiliations:** 1 Department of Orthopedic Surgery, Oakland University William Beaumont School of Medicine, Rochester, USA; 2 Department of Orthopedic Surgery, Centro Médico Jardins, Aracaju, BRA; 3 Department of Medicine, Oakland University William Beaumont School of Medicine, Rochester, USA; 4 Department of Orthopedic Surgery, Maimonides Medical Center, New York, USA; 5 Department of Orthopedic Surgery, Texas Tech University Health Sciences Center, El Paso, USA; 6 Department of Orthopedic Surgery, University of New Mexico Health Sciences Center, Albuquerque, USA

**Keywords:** mass, case series, first web space, tumor, hand

## Abstract

Introduction: Hand masses are fairly common. While most of these masses are either ganglion cysts or benign tumors, masses in the first web space are not rare, and they may in fact represent a variety of lesions. These include both benign and malignant tumors, metastases, or congenital and anomalous structures, and may involve nerves, vascular structures, connective tissue, and joints.

Methods: In this retrospective case series, data on 12 cases of first dorsal web space hand mass treated at our center over a period of five years were collected and analyzed.

Results: Twelve consecutive patients presenting with a first dorsal web space hand mass over a period of five years were reviewed. This represented a group of nine females and three males, with a mean age of 53 years (range = 16-70 years). Seven patients had a mass on the right side and five on the left side. The surgical approach to resect the mass in all 12 patients was dorsal. The most common diagnosis was ganglion cyst (50%), followed by lipoma (25%) and aneurysm (16.6%). There was one case of eccrine spiradenoma.

Conclusion: First dorsal web space hand masses can encompass multiple different pathologies, and the first web space has an intricate anatomy. Both of these factors mandate a careful approach that includes meticulous preoperative planning with appropriate advanced imaging studies, which helps to make the surgical procedure more efficient and accurate.

## Introduction

Clinical anatomy

Radial Artery and Radial Sensory Nerve

An artery to the dorsal ridge of the scaphoid is a direct branch from the radial artery in 75% of cases and it originates from the radiocarpal or intercarpal artery in 25% of cases [1]. Numerous anomalies and variations of the radial artery in the anatomical snuffbox area and the dorsal first web space have been reported, including tortuous configurations, hypoplasia, and the absence of the radial artery. It may also course superficial to the extensor tendons of the thumb (instead of deep to these structures) and may be confused with a superficial vein [2]. The Allen test would be invaluable to preoperatively evaluate the competency of the major vessels to the hand. The radial sensory nerve arises between the tendons of the brachioradialis and extensor carpi radialis longus 8.6 cm proximal to the radial styloid, piercing the forearm fascia 6 cm from the radial styloid. Approximately six sensory branches cross the wrist joint and at least three of these branches overlie the first web space and thus are liable to damage during surgical dissection in this region [3].

Tendons, Muscles, and Joints

Variations of the extensor tendons of the hand are common, and the vast majority of those variations are asymptomatic throughout the life of the individual. However, they may present as a dorsal mass. These anomalous muscles are closely related to the extensor indicis proprius tendon and may overlie the first web space [4]. Variations of the adductor pollicis have also been observed. The two heads of the muscle may vary in size and can be split into additional bellies. The transversus manum muscle is an anomalous muscle closely related to the adductor pollicis. It arises from the palmar metacarpophalangeal ligaments and connects to the base of the thumb proximal phalanx or its vicinity. Anomalies of the interosseous muscles also have been reported, including the absence or additional presence of muscle bellies [2]. Several joints exist in the vicinity of the first web space, and they may be the possible origin of ganglions. Of notice are the scaphotrapezial, trapezial-first metacarpal, trapezial-second metacarpal, radioscaphoid, and scapholunate joints [5,6].

The purpose of this article is to present 12 cases of first dorsal web space hand masses collected over a period of five years from our practice and to discuss a diagnostic approach to include preoperative history, physical examination, and appropriate imaging studies in conjunction with an anatomical understanding of this area.

## Materials and methods

Study design

Twelve consecutive patients presenting to the University of New Mexico Hospital with a first dorsal web space hand mass over a period of five years were reviewed to formulate this retrospective case series.

Inclusion and exclusion criteria

Patients presenting with a first dorsal web space hand mass were included in the study. Any other hand masses were excluded.

Data collection

Patient information obtained from the charts included clinical symptoms and signs, imaging studies, preoperative and postoperative diagnosis, surgical findings, pathology report, and follow-up data regarding the recurrence of the lesion and patient outcomes.

## Results

Twelve consecutive patients presenting with a first dorsal web space hand mass over a period of five years were reviewed. This represented a group of nine females and three males, with a mean age of 53 years (range = 16 to 70). Seven patients had a mass on the right side and five on the left side. The surgical approach to resect the mass in all 12 patients was dorsal. The most common diagnosis was ganglion cyst (50%), followed by lipoma (25%) and aneurysm (16.6%). There was one case of eccrine spiradenoma.

**Figure 1:**
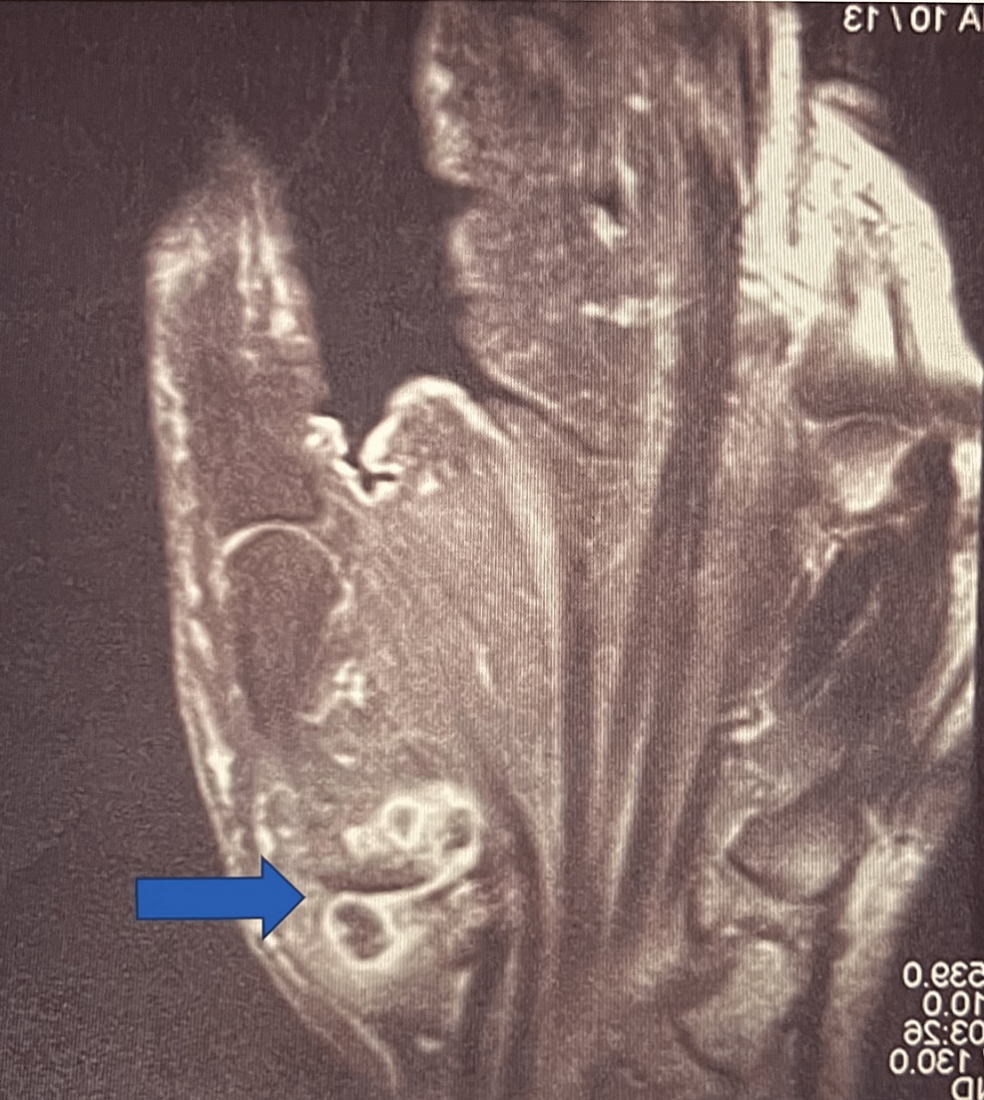
MRI of a right first carpometacarpal joint ganglion of a 60-year-old patient. The multiloculated complex ganglion is marked by the blue arrow.

Four out of six patients presenting with ganglions complained of a painful mass, and the remaining patients reported no pain. All the ganglions were transilluminated and the diagnosis was made on clinical grounds. MRI was performed on three of those patients and was useful in determining the origin of the ganglion. During surgery, it was observed that the most common origin of the ganglion was from the second metacarpal-trapezoid joint (3/6), followed by the first carpometacarpal joint (2/6) (Figure 1), and the scapholunate joint (1/6) (Table 1).

**Table 1:** Clinical data and outcomes of 12 patients with mass in the first dorsal web space of the hand n/r: no recurrence.

Number	Age/sex	History	X-ray	MRI	Surgical findings/size	Pathology	Follow-up
1	55/F	Asymptomatic mass/4 years	Normal	Lipoma	Lipoma 1^st^ web and palm/6 x 3 cm	Lipoma	3 years, n/r
2	62/F	Mass/numbness/2 years	Normal	Lipoma	Lipoma 1^st^ web/3 x 3 cm	Lipoma	8 months, n/r
3	50/M	Asymptomatic mass/3 years	Normal	Atypical lipoma/liposarcoma	Lipoma first web/4 x 3 cm	Lipoma	6 months, n/r
4	50/F	Painful mass/3 years	Normal	None	Ganglion, 2^nd^ metacarpotrapezoid joint/1 cm	Ganglion	1.5 years, n/r
5	63/F	Recurrent mass	Normal	Multilobed nonspecific mass	Giant cell tumor/1.5 x 1 cm	Eccrine spiradenoma	8 months, n/r
6	60/F	Asymptomatic mass/2 years	Normal	None	Ganglion, 2^nd^ metacarpotrapezoid joint/2 cm	Ganglion	4 years, n/r
7	60/F	Asymptomatic mass/2 years	Normal	Ganglion 1^st^ carpometacarpal joint	Ganglion 1^st^ carpometacarpal joint/3 cm	Ganglion	3 years and 2 months, n/r
8	31/M	Painful mass/3 years	Normal	Ganglion 2^nd^ metacarpotrapezoid joint	Ganglion 2^nd^ metacarpotrapezoid joint/1 cm	Ganglion	1 year and 5 months, n/r
9	35/M	Painful mass 2 years	Normal	Ganglion scapholunate joint	Ganglion scapholunate joint/2 cm	Ganglion	3 years, n/r
10	55/F	Painful mass, 5 years	Osteoarthritis 1^st^ carpometacarpal joint	None	Ganglion 1^st^ carpometacarpal joint, 1.5 cm	Ganglion	2 years, n/r
11	16/F	Penetrating injury, pulsatile mass	Normal	Pseudoaneurysm radial artery	Pseudoaneurysm radial artery, 2 x 1 cm	Pseudoaneurysm	6 months, n/r
12	70/F	Pulsatile mass, diabetes mellitus, atherosclerosis	Normal	Aneurysm/radial artery	Aneurysm/radial artery, 2 x 2 cm	Aneurysm	2 years and 8 months, n/r

**Figure 2:**
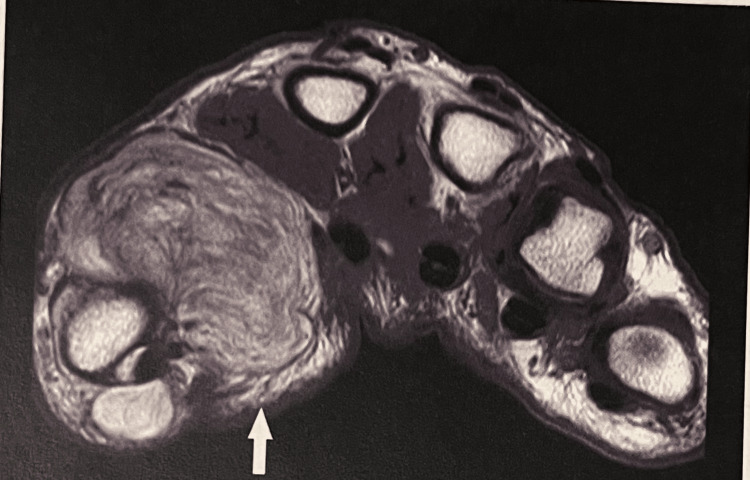
MRI of a first web space lipoma (white arrow).

One of the patients presenting with the first carpometacarpal joint ganglion had X-ray findings of osteoarthritis in that joint. None of the patients whose diagnosis was a lipoma complained of pain. However, one of them (1/3) complained of paresthesia along the median nerve distribution. The MRI was diagnostic in all those patients (Figure 2).

**Figure 3:**
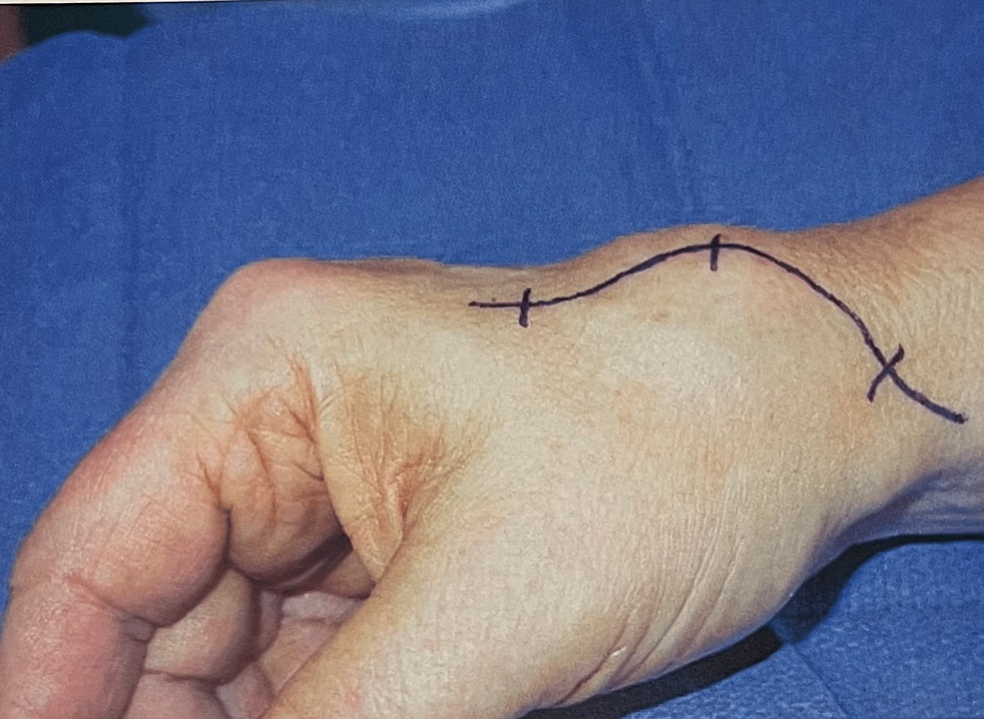
Clinical picture of a patient with a first dorsal web space pseudoaneurysm.

**Figure 4:**
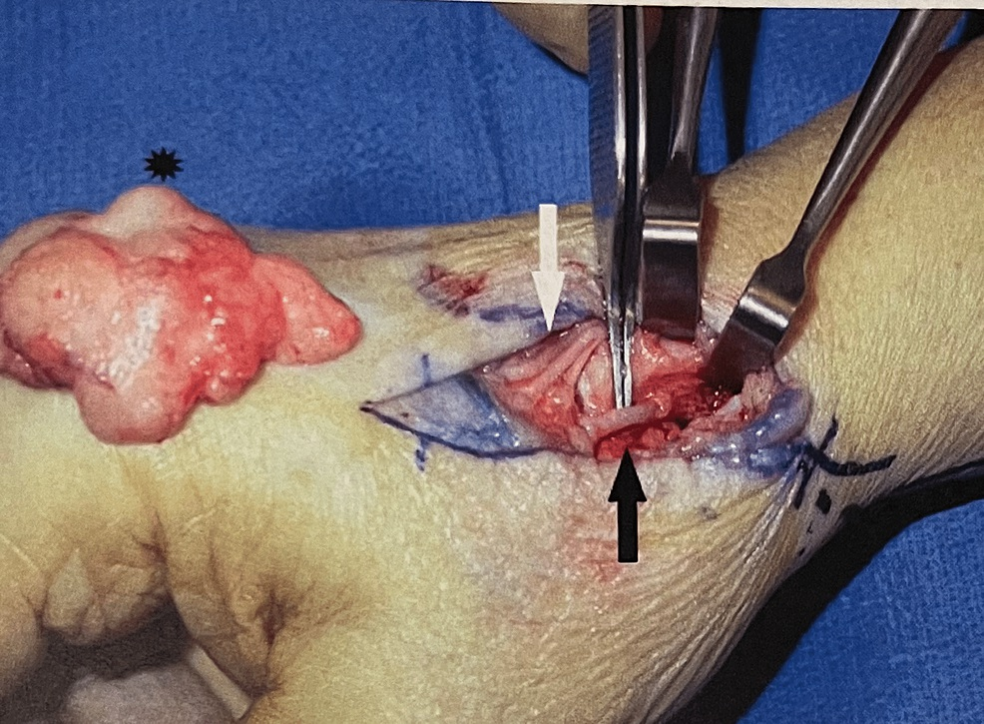
After excision of the pseudoaneurysm (black asterisk). The black arrow indicates the dorsal branch of the radial artery. The white arrow indicates three sensory branches of the radial nerve.

The two patients diagnosed with radial artery aneurysms presented with a painless pulsatile mass. One of them had a history of penetrating injury (pseudo aneurysm) (Figures 3, 4), and the other had generalized atherosclerosis (true aneurysm).

The patient diagnosed with eccrine spiradenoma had a history of a hand mass resection four years previously. At that time, no pathologic studies were performed. The recurrent hand mass was painless. MRI was performed and was nonspecific. Our preoperative diagnosis was a giant cell tumor. The MRI, although nondiagnostic due to the nonspecific appearance of the lesion, excluded a vascular origin, a ganglion, or a fatty lesion (lipoma), and pointed to the fact that the lesion did not appear to originate from the adjacent tendon sheath.

The mean follow-up of these patients was one year and nine months. There was no local recurrence of the mass in any of the patients at the latest follow-up.

## Discussion

The differential diagnosis of a first dorsal web space mass may be extensive and our cases may be unrepresentative of the true occurrence of each of these lesions. We did a literature review and found that there are case reports describing one or two cases of first dorsal web space masses, which makes our case series the largest on this subject to our knowledge [7-11].

A good history and physical examination complemented by imaging studies that may include both routine plain radiographs and MRI studies are usually conducive to the correct diagnoses [12,13]. We feel strongly that preoperative planning makes for a more efficient operative experience for both the surgeon and the patient. In this series, only one case was not correctly diagnosed preoperatively (eccrine spiradenoma).

Ganglion cysts are the most common hand masses and corresponded to half the cases in our series. The ganglion may, however, originate from multiple sites, including the radioscaphoid and scapholunate interval, scaphotrapezial joint, and first and second carpometacarpal joints [5,14]. Ganglions may also arise from arthritic joints, as was the case in one of our patients (case 10). Clues to a ganglion cyst include a mass that fluctuates in size, is compressible, and usually transilluminates. Although clinical diagnosis is usually enough to achieve the correct diagnosis, we feel that, particularly in this region, further work-up is warranted to locate the joint from where the ganglion arises and determine its relation to adjacent structures, which are fundamental for surgical planning. In the patients for whom we ordered preoperative MRI, the surgical procedure was more straightforward.

Lipomas were the second most common tumors that we encountered in this group of patients, although they occur infrequently in the hand and wrist area [15]. They usually originate in the deep palmar space and spread to the first dorsal web space. Those masses are usually nontender; however, some patients may complain of paresthesia due to nerve compression. Physical examination most commonly reveals a soft, nontender mass that does not transilluminate. X-rays may be unremarkable or demonstrate a radiolucent area. MRI imaging appears to be the best imaging modality and is usually diagnostic [12,13,16]. It shows a mass whose signal intensity parallels that of the subcutaneous fat on all pulse sequences. Fat typically produces a high signal with T1-weighted imaging, an intermediate to high signal on T2-weighted images, and a low signal with fat-suppressed images. Again, dissection in this area is tedious and the knowledge of the topography of the tumor and nearby vascular and nerve structures is invaluable.

In this series of patients, we encountered two cases of radial artery aneurysms. The diagnosis is usually straightforward when a pulsatile mass over an artery is found. It is important to differentiate a true aneurysm from a false aneurysm (pseudoaneurysm) [17].

False aneurysms can occur secondary to penetrating trauma of the vessel wall with subsequent hemorrhage (case 11). The lumen of the false vessel is in continuity with the true vessel; however, it is devoid of an endothelial lining. True aneurysms occur after injury to the vessel that permits gradual dilatation of the vessel. Whereas false aneurysms usually occur after penetrating injuries, true aneurysms are the result of blunt or repetitive trauma. Atherosclerotic arterial disease is a predisposing factor for aneurysms, since it weakens the vessel wall, allowing gradual vessel dilation to occur, especially in the setting of high blood pressure (case 12). The natural history of both is characterized by slow progression, leading to thrombosis or embolism [17]. MRI without angiography was used in both patients for diagnosis and surgical planning, avoiding the inconvenience of invasive studies.

The occurrence of one case of eccrine spiradenoma emphasizes the fact that virtually any tumor can occur in this area. This tumor of sweat gland derivation most commonly occurs in the trunk and head. It is considered a rare tumor in the hand with only a few cases reported in the English literature [18,19]. The correct preoperative diagnosis is rarely made because of its infrequent occurrence and the lack of any clinical distinctive features. Recurrence is uncommon and is considered to be due to inadequate initial resection, thus emphasizing the need for preoperative MRI studies to help define the extent of the soft tissue mass.

## Conclusions

In conclusion, first dorsal web space hand masses can encompass multiple different pathologies, and the first web space has an intricate anatomy. Both of these factors mandate a careful approach that includes meticulous preoperative planning with appropriate advanced imaging studies, which helps to make the surgical procedure more efficient and accurate. If there is a question regarding the diagnosis prior to surgery, a fine needle aspiration biopsy can be considered. Other diagnostic studies that can be considered include an ultrasound.
